# Structural and Thermodynamic Properties of Selective Ion Binding in a K^+^ Channel

**DOI:** 10.1371/journal.pbio.0050121

**Published:** 2007-05-01

**Authors:** Steve W Lockless, Ming Zhou, Roderick MacKinnon

**Affiliations:** 1 Laboratory of Molecular Neurobiology and Biophysics, The Rockefeller University, New York, New York, United States of America; 2 Howard Hughes Medical Institute, The Rockefeller University, New York, New York, United States of America; University of Texas Austin, United States of America

## Abstract

Thermodynamic measurements of ion binding to the Streptomyces lividans K^+^ channel were carried out using isothermal titration calorimetry, whereas atomic structures of ion-bound and ion-free conformations of the channel were characterized by x-ray crystallography. Here we use these assays to show that the ion radius dependence of selectivity stems from the channel's recognition of ion size (i.e., volume) rather than charge density. Ion size recognition is a function of the channel's ability to adopt a very specific conductive structure with larger ions (K^+^, Rb^+^, Cs^+^, and Ba^2+^) bound and not with smaller ions (Na^+^, Mg^2+^, and Ca^2+^). The formation of the conductive structure involves selectivity filter atoms that are in direct contact with bound ions as well as protein atoms surrounding the selectivity filter up to a distance of 15 Å from the ions. We conclude that ion selectivity in a K^+^ channel is a property of size-matched ion binding sites created by the protein structure.

## Author Summary

The exquisite selectivity of potassium ion (K^+^) channels in cellular membranes allows them to pass K^+^ ions while restricting the closely related sodium (Na^+^) ions, and thereby maintain the electrical potential across cellular membranes. In this study, we address the fundamental question: how does the K^+^ channel discriminate between K^+^ and Na^+^ ions? Past studies have relied on nonequilibrium measurements of ionic current flow. We measured heat exchange associated with ion binding to the channel under equilibrium conditions and determined crystal structures of ion-bound and ion-free forms of the channel. By studying a series of alkali metal and alkaline earth cations, we documented the effect of varying systematically the ionic charge and radius, and we discovered that the K^+^ channel recognizes an ion's size rather than its electric field strength. By analyzing the structures, we show that the channel's ability to recognize an ion's size is a function of protein atoms that are both near to and far away from the ion binding sites. This study opens a new window into ion selectivity in channels and also contributes to our expanding knowledge of the emerging role of long-range interactions in ligand recognition.

## Abstract 3

Ion selectivity of the KcSA K^+^ channel is assessed through a combination of thermodynamic measurements of ion binding and structural analysis of ion-bound and ion-free conformations.

## Introduction

**Figure 1:**
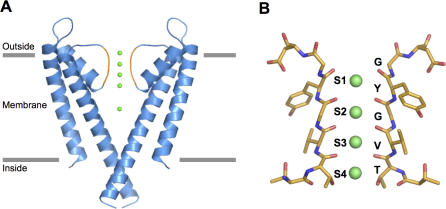
K^+^ Ion Binding Sites within the KcsA K^+^ Channel (A) A ribbon representation of KcsA with two of the four subunits is shown; the subunits closest to and furthest from the viewer are removed for clarity. K^+^ ions (in green) are located within the selectivity filter (orange) and in the water-filled cavity. The gray lines indicate the presumed interior and exterior membrane boundaries. (B) A stick representation of the selectivity filter containing four K^+^ ion-binding sites (S1–S4). Each K^+^ ion-binding site is composed of eight oxygen atoms made from the K^+^ channel TVGYG signature sequence. The figure was made using PyMOL [33].

Potassium channels conduct K^+^ ions at nearly diffusion-limited rates, and at the same time, they prevent Na^+^ ions from conducting [1]. The ability to distinguish between K^+^ ions (Pauling ionic radius 1.33 Å) and Na^+^ ions (Pauling ionic radius 0.95 Å) occurs within a segment of the pore known as the selectivity filter (Figure 1A). Inside the selectivity filter, K^+^ ions are coordinated by oxygen atoms from the protein, which replace the water molecules that normally surround a hydrated ion (Figure 1B) [2,3].

Several different operational definitions of ion selectivity or relative permeability exist in the electrophysiology literature [4,5]. Ion selectivity is sometimes quantified by comparing the conduction rates of different ions, which is analogous to defining the substrate specificity of an enzyme by measuring the catalytic rate with different substrates [6,7]. Other times, ion selectivity is defined by a measure of the degree to which one ion will conduct relative to another (out compete) when both ions are present in solution at the same time, akin to a substrate competition assay. These different definitions are not related in any simple manner, and their physical interpretation (in terms of the interaction energy between an ion and the channel) depends on specific details of the conduction mechanism.

Because ion channels generally operate far from equilibrium, it seems reasonable to define ion selectivity as electrophysiologists have, by using the nonequilibrium conditions under which channels normally function. On the other hand it should be possible to characterize ion selectivity under equilibrium conditions if one had a method for quantifying the affinities of different ions for the channel. Selectivity defined in such a way would depend not on the kinetic details of the conduction mechanism, but rather on the energy difference between the ion-bound and unbound states, which should be directly related to the atomic structures of these states.

In this study, we have measured ion binding to a K^+^ channel under equilibrium conditions using isothermal titration calorimetry (ITC), and we have determined the structures of ion-bound and ion-free forms of the channel. These experiments permit us to correlate structural and thermodynamic properties of selective ion binding. Our aim is to understand which property of an ion (i.e., size or charge density) is recognized by the channel and which properties of the channel create an energetically favorable match for the ion.

## Results

### Alkali Metal Ion Binding to KcsA Using ITC

**Figure 2:**
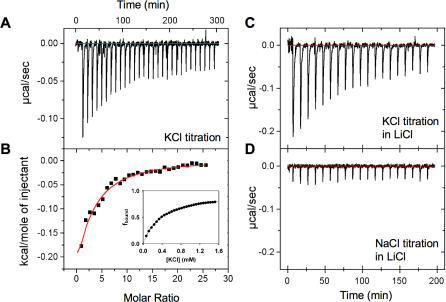
ITC Titration of Alkali Metal Cations Binding to KcsA (A) A KCl solution is titrated into a solution containing KcsA. Each downward deflection corresponds to one injection. The area represents the heat exchanged. (B) The total heat exchanged during each injection is fit to a single-site binding isotherm with *K*_D_ and Δ*H*° as independent parameters, where *K*_D_ = 0.41 mM and Δ*H*° = −1.4 kcal/mol. Similar values for *K*_D_ and Δ*H*° are obtained using different protein concentrations varied over a 5-fold range. The inset shows the same data represented as the fraction of ion-bound KcsA as a function of [KCl]. (C) A KCl solution titrated into KcsA in a background solution of LiCl instead of NaCl (as is used in Figure 2A) shows heat from ion binding. (D) A NaCl solution titrated into KcsA in the background of LiCl shows only the heat of diluting NaCl without binding to KcsA.

Figure 2A shows an ITC experiment in which a solution containing KCl is titrated into a solution containing Streptomyces lividans K^+^ channels (KcsA) in NaCl. Each downward deflection results from the heat of diluting KCl from the injection syringe solution into the reaction chamber plus the net heat of transfer of K^+^ ions from solution to the channel. There are several points to note about this titration. First, there is a gradual decrease in the amount of heat liberated upon successive KCl injections until a constant level is eventually reached. This pattern suggests that the channel's binding sites are becoming saturated so that the final injections represent only the heat of diluting the concentrated KCl solution into the protein solution. In control experiments in which the channel protein is not present in the reaction chamber, one observes only this constant heat of dilution. Second, the binding reaction is exothermic, which means the transfer of K^+^ ions from water to the channel is enthalpically favored. And third, the rate of heat release (and thus K^+^ binding) is relatively slow, as evidenced by the longer duration of the deflections in the beginning compared to the end of the titration.

In Figure 2B, the heat transfer associated with each injection is plotted as a function of the ligand (K^+^)-to-protein concentration ratio and fit to an equation that incorporates the enthalpy and affinity of a single K^+^ ion binding event (see Materials and Methods) [8]. The fit (solid line) corresponds to stoichiometry of binding *n* = 1, dissociation constant *K*_D_ = 0.41 mM, and enthalpy Δ*H*° = −1.4 kcal/mol. The inset contains the same data plotted in a more familiar fashion: the fraction of total heat liberated (the transient component due to K^+^ binding) as a function of the K^+^ concentration. A detailed description of the ion binding model, which is based on a combination of ITC and x-ray crystallographic data, is given in Materials and Methods.

When the K^+^ titration experiment was repeated using LiCl as the background electrolyte (Figure 2C) instead of NaCl (Figure 2A), the outcome was similar: heat is liberated upon addition of K^+^. By contrast, heat is not liberated upon addition of Na^+^, which has a Pauling radius that is smaller than that of K^+^ (Figure 2D). The larger alkali metal ions Rb^+^ (Pauling radius 1.48 Å) and Cs^+^ (Pauling radius 1.69 Å) are like K^+^, binding to the channel with estimates for *K*_D_ and Δ*H*° given in Table 1.

**Table 1:**
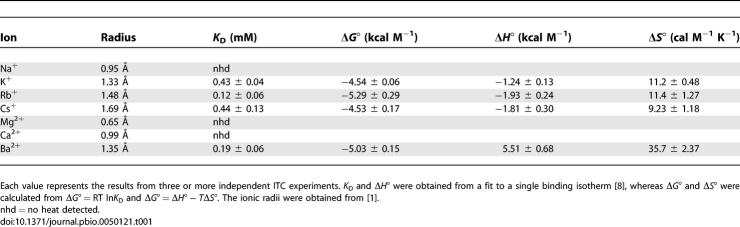
Thermodynamic Parameters Obtained for Ion Binding to KcsA

In electrophysiological experiments, K^+^ channels are known to discriminate strongly against smaller alkali metal ions Li^+^ and Na^+^ but permit the larger Rb^+^ and Cs^+^ ions to conduct. We therefore observe a correlation between electrophysiological permeability and heat liberation by ITC.

### Filter Conformational Change Underlying Ion Binding

**Figure 3:**
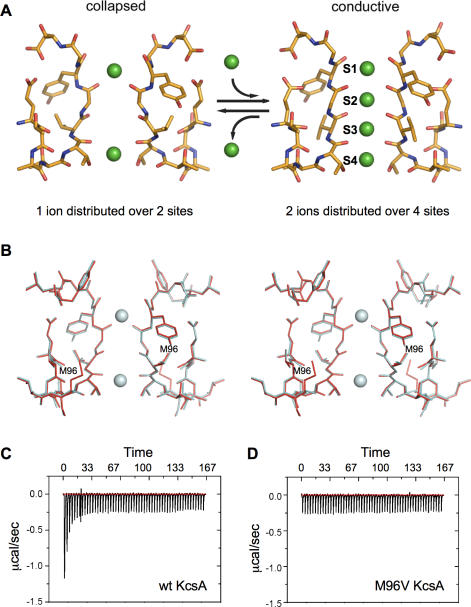
A Conformational Change Underlies the Enthalpy of Ion Binding (A) The KcsA selectivity filter exists in two conformations that depend on whether one or two ions are bound. The collapsed conformation (left) has one ion distributed over two sites, whereas the conductive conformation (right) has two ions distributed over four sites. Amino acids E71 to D80 are shown in a stick diagram (from PDB 1K4D and PDB 1K4C) encompassing the selectivity filter (T75-G79) and surrounding residues. (B) This stereo-view compares the KcsA-M96V structure (gray) to the KcsA-wt collapsed structure (red) and shows that the M96V mutant remains in the collapsed conformation even at 300 mM KCl. (C) An ITC titration of wild-type KcsA with 400 mM KCl in the syringe shows most of the ion binding occurs within the first few injections. (D) An ITC titration of the M96V mutant with KCl shows no K^+^ binding.

Crystal structures of KcsA show that its selectivity filter can exist in two distinct conformations associated with low and high concentrations of K^+^ (Figure 3A) [2,9]. In solutions containing less than 5 mM KCl and 150 mM NaCl, the filter adopts a nonconductive conformation, which is pinched closed (Figure 3A, left). Crystallographic occupancy studies show that in this conformation, ions bind at the ends of the filter (sites 1 and 4), with up to a single K^+^ ion distributed over these two sites [9]. As the concentration of K^+^ in the crystallization solution is increased, a second ion enters, in association with a conformational change of the filter to the conductive form (Figure 3A, right). In the conductive form, two K^+^ ions are distributed over four sites, each with approximately half occupancy [9,10]. The crystallographic data have thus demonstrated that the entry of a K^+^ ion (into the middle of the filter, sites 2 and 3) is associated with a specific conformational change of the selectivity filter.

Is the heat transfer associated with ion binding correlated with the filter conformational change? To address this question, we studied a mutant channel, M96V, which crystallographically remains in the nonconductive conformation at K^+^ concentrations up to 300 mM K^+^ (Figure 3B). In the ITC assay of this mutant channel, no detectable heat is liberated upon addition of K^+^ (Figure 3C and 3D). This mutant channel in every other respect appears like the wild-type channel at low concentrations of K^+^; the only difference is that as the K^+^ concentration is raised, the channel fails to undergo the nonconductive-to-conductive conformational change (and thus fails to bind a K^+^ ion at sites 2 and 3). These observations suggest that the heat measured in the ITC assay on the wild-type channel is liberated when the filter undergoes its conformational change and binds a K^+^ ion at sites 2 and 3 (Figure 3A).

**Figure 4:**
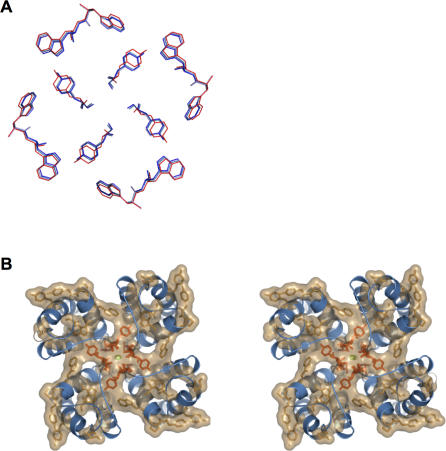
Propagated Residue Displacements Correlate with Ion Binding and Co-Evolving Positions (A) A comparison of the collapsed and conductive conformations of KcsA is shown. The collapsed Na^+^-bound structure is shown in red, whereas the conductive K^+^-, Rb^+^-, or Cs^+^- bound structures are shown in various shades of blue. This image is a slice through the S2 ion-binding site as viewed from the extracellular side of the membrane looking down the pore. (B) A stereo-view of the highly conserved selectivity filter (orange) and conserved plus co-evolving (yellow) positions of K^+^ channels is shown as a stick representation mapped onto the ribbon representation (blue) of KcsA. The brown surface envelops the conserved and co-evolving positions. This view is from the extracellular side of the membrane looking down the pore.

The coupling of an ion-binding event to a conformational change of the filter can explain why the rate of ion binding is slow in the ITC assay. The readjustment of protein atoms occurs up to a distance of 15 Å from the ion pathway (Figure 4A). The atomic displacements are essentially the same whether K^+^, Rb^+^_,_ or Cs^+^ ions bind (Figure S1). It is undoubtedly significant that the conformational change involves amino acids that are highly coupled to one another in an analysis of sequence co-evolution (Figure 4B). We suspect that these amino acids have been constrained by natural selection to enable the filter to bind K^+^ at sites 2 and 3, but not Na^+^.

The data discussed so far support the following conclusions. Alkali metal ions K^+^, Rb^+^, and Cs^+^ can bind to the internal sites (2 or 3) of the selectivity filter. Their binding is associated with a specific conformational change of the filter to a conductive form and an exchange of heat with the environment. Na^+^ (and presumably Li^+^) does not bind to sites 2 or 3, stabilize the conductive conformation, or liberate heat.

### Alkaline Earth Ion Binding to KcsA Using ITC

**Figure 5:**
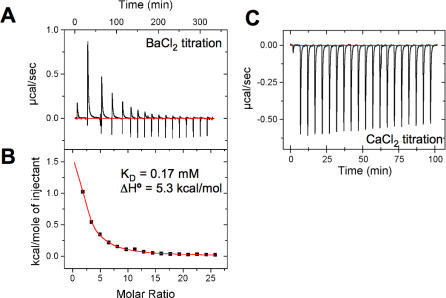
ITC Titration of Alkaline Earth Metal Cations Binding to KcsA (A) A BaCl_2_ solution is titrated into a solution containing KcsA. (B) The total heat exchanged is fit to a single binding isotherm with *K*_D_ and Δ*H*° as independent variables. (C) A CaCl_2_ solution titrated into a solution containing KcsA shows no ion binding.

An obvious distinction between the different alkali metal cations is their atomic radius. By studying the alkali metal ion series, we have documented how the ionic radius affects the ability of a +1 ion to interact with the selectivity filter. We observed a size cut-off between Na^+^ and K^+^. Next, we investigated a series of different sized alkaline earth (+2) cations. Figure 5A shows an ITC titration with BaCl_2_ into a KcsA solution with NaCl as the background electrolyte. The fit corresponds to *K*_D_ = 0.17 mM and Δ*H*° = +5.3 kcal/mole (Figure 5B, solid line). By contrast, no measurable heat of ion binding to KcsA is detected with either Ca^2+^ (Figure 5C) or Mg^2+^ (unpublished data). The ionic radius of Ba^2+^ (1.35 Å) is very close to that of K^+^ (1.33 Å), whereas the radii of Mg^2+^ (0.61 Å) and Ca^2+^ (0.99 Å) are close to that of Li^+^ (0.60 Å) and Na^+^ (0.95 Å), respectively.

For Ba^2+^_,_ Δ*H*° is positive (unfavorable), but a large favorable entropy term, Δ*S*°, results in a free energy change Δ*G*° for binding that is actually slightly more favorable for Ba^2+^ than for K^+^ (Table 1). The important point, however, is that Ba^2+^ binds to the filter, whereas Ca^2+^ and Mg^2+^ do not. This means the size cut-off for selective binding of +2 cations occurs between Ca^2+^ (radius 0.99 Å), which does not bind, and Ba^2+^ (radius 1.35 Å), which binds. This is the same size cut-off that is observed in the +1 alkali metal cation series. The ability of Ba^2+^ to bind to the selectivity filter as measured using ITC is in good agreement with electrophysiological studies on the interaction of Ba^2+^ with K^+^ channels [11,12].

**Figure 6:**
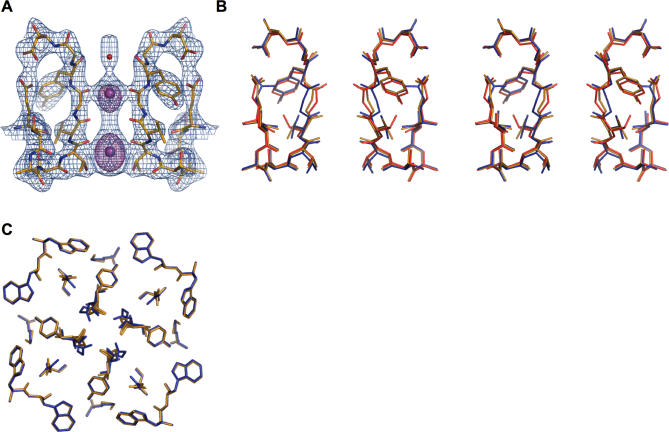
Structure of the Selectivity Filter with Ba^2+^ Bound Inside (A) A 2Fo-Fc electron density map (colored in blue and contoured at 2 sigma) of the selectivity filter region show two diagonally opposing subunits. Ba^2+^ ions (in magenta) and H_2_O (in red) are shown as spheres within the filter. The anomalous difference density map is shown in magenta as a fine mesh (contoured at 10 sigma) and was used to identify the Ba^2+^ ions. (B) A stick representation shows a comparison between the selectivity filter of the Ba^2+^ bound structure (yellow) with the collapsed (red) and conductive (blue) K^+^ bound structures. (C) Comparing the Ba^2+^- bound (yellow) and conductive conformation (blue) structures shows that the Ba^2+^- bound structure adopts the conductive conformation at distant sites along the plane of aromatic amino acids surrounding the selectivity filter.

Does Ba^2+^ binding require the protein conformational change within the filter? An earlier structure of a Ba^2+^ complex of KcsA could not address this question because it was determined at low resolution (5 Å) [13]. We have determined a Ba^2+^ structure with crystals that diffract to 2.7 Å. A 2Fo-Fc electron density map calculated after omitting the selectivity filter and ions is shown (Figure 6A, blue mesh). An x-ray anomalous signal defines unequivocally the location of Ba^2+^ ions in the filter at site 4, as determined previously at lower resolution [13], and near site 2 at the center of the filter (Figure 6A, magenta mesh). Figure 6B shows in superposition the structures of the Ba^2+^ complex (yellow), the conductive K^+^ complex (blue), and the nonconductive (low K^+^) KcsA structure (red). At amino acid glycine 77, whose carbonyl oxygen atom coordinates the Ba^2+^ ion near site 2, the carbonyl carbon resides at a location in between the conductive and nonconductive conformations. Everywhere else, protein atoms of the Ba^2+^ complex adopt the conductive structure, as can be seen by the side chain rotamer of valine 76 (Figure 6B) and the superposition of aromatic amino acids surrounding the filter for the Ba^2+^ and conductive K^+^ structures (Figure 6C). Thus, size selective binding of +2 cations (i.e., Ba^2+^ and not Ca^2+^ or Mg^2+^) depends on the ability of the filter to adopt the conductive conformation, just as for monovalent cations.

## Discussion

By studying a series of ions with different atomic radii, we observe a dependence on the ability of ions to bind to the interior of the selectivity filter. Among the alkali metal ions, K^+^, Rb^+^ and Cs^+^ bind and liberate heat, whereas Na**^+^** does not. Among the alkaline earth ions, Ba^2+^ binds, whereas Ca^2+^ and Mg^2+^ do not. The binding events are correlated with specific protein conformational changes, which enable a selected ion to bind to sites 2 or 3 of the selectivity filter.

The thermodynamic measurements of ion binding correlate in a qualitative manner with the ability of K^+^, Rb^+^_,_ and Cs^+^ to conduct through K^+^ channels and the ability of Ba^2+^ to enter the pore and cause blockage. In quantitative detail, however, the “permeability sequence” determined electrophysiologically is not the same as the binding affinity sequence determined in this study. The permeability of K^+^ for most K^+^ channels is slightly greater than that of Rb^+^, which is greater than that of Cs^+^, whereas the permeability of Na^+^ and Li^+^ is extremely small [5,14]. Ba^2+^ blocks K^+^ conduction but itself, conducts poorly. In the setting of these significant electrophysiological differences between the ions, the equilibrium binding affinities for K^+^, Rb^+^, Cs^+^_,_ and Ba^2+^ are fairly similar to each other (Table 1). This difference between permeability (large variation) and affinity (small variation) is not a discrepancy, because permeability and binding affinity are two different entities. Permeability is a nonequilibrium quantity reporting how well an ion conducts, which depends on the depth of energy wells and the height of energy barriers encountered by ions as they diffuse through the pore. Binding affinity is an equilibrium quantity and depends on the path-independent energy difference between the reactants (channel + ion) and product (complex).

How does the ion-binding event that is the focus of this study relate to ion conduction? The affinity of the K^+^ ion for the filter (*K*_D_ ∼ 0.5 mM) precludes the possibility that transport is simply the forward and backward steps of the binding reaction. The *K*_D_ places an upper limit on the rate at which an ion can dissociate (and thus be transported) because *k*_off_ = *k*_on_ × *K*_D_. For example, if *k*_on_ were diffusion limited and had a rate of 10^8^ M^−1^s^−1^; then *K*_D_ would limit *k*_off_ (and therefore the conduction rate) to about 10^5^ ions s^−1^. Thus, we assume that when the filter is conducting ions at a high rate (up to about 10^8^ ions s^−1^), it remains in its conductive conformation as ions flow through. This assumption is compatible with the slow rates of ion binding in the ITC assay: once binding has occurred in association with the conformational transition to the conductive form, which may be slow, K^+^ can then diffuse through at a high rate.

The above discussion implies that the ITC experiments record an ion-binding event (associated with a protein conformational change) that is not a step in the conduction process, but rather one that puts the filter into its conductive conformation. Nevertheless, the specificity of this binding event (K^+^ and not Na^+^ mediated) appears to be deeply rooted in the ability of the filter to select the correct ion by stabilizing a specific (conductive) structure in response to ions that satisfy a certain criterion.

What is the criterion or physical property of an ion that allows it to enter the filter and stabilize the conductive conformation? Obviously ion size is important. Is this because the binding site(s) favor an ion for its size or for the strength of the electric field near its surface? The electric field strength, which is proportional to the charge density, is of course a function of an ion's size. By studying ions with different ionic radii and different charges, we find that the ability to bind and stabilize the conductive conformation depends on the same cut-off radius for both +1 and +2 cations. If the ability of an ion to bind depended on field strength as a “primary” property, then Na^+^ and Ba^2+^ should be similar. This is not the case: K^+^ and Ba^2+^, which have different electric field strengths at their surface but nearly the same size, bind with similar affinities. This result informs us that an ion's size, not simply through the effect of size on field strength, is an important criterion for selectivity in K^+^ channels.

How does the K^+^ channel detect the size of an ion? The crystal structures in different ionic solutions show that the selectivity filter has the potential to adopt two distinct, well-defined conformations that we call conductive and nonconductive. The conductive conformation is associated with ion-binding sites that are each formed by eight carbonyl oxygen atoms (sites 1–3) surrounding a “hole” into which a K^+^ ion snugly fits and satisfies its preference for six to eight coordinating oxygen atoms [15]. A very slight rotation of the carbon–oxygen bond permits larger ions such as Rb^+^ and Cs^+^ to fit snugly into these same sites without perturbing the conductive conformation. The sites in the conductive conformation are not compatible with the smaller radius of Na^+^: given only Na^+^ in solution, we observe experimentally that the channel enters the nonconductive conformation rather than binding Na^+^ at sites 2 and 3 (i.e., the nonconductive channel is more stable than a Na^+^-bound conductive channel). Thus, in answering the question how does the K^+^ channel detect the size of an ion, we are led to conclude that the conductive conformation is associated with ion-binding sites that match the size of K^+^ but are too large for Na^+^. The channel is thus able to compensate for the dehydration energy of K^+^ but not Na^+^.

It is the conductive conformation of the channel rather than the transition from nonconductive to conductive that confers selectivity. After all, when the nonconductive conformation is prevented by mutation, the channel still binds K^+^ selectively over Na^+^ [16]. However, the crystallographically observed transition is fortuitous, because it reveals to us the regions of the channel that are responsible for creating the binding sites as they are in the conductive conformation. The filter atoms and the surrounding protein atoms are important for creating the selective ion-binding sites (Figure 4).

The above data and conclusions are in agreement with the general ideas behind the “snug fit” hypothesis of Armstrong and Hille for ion binding in a selective channel [17,18]. The data are not in agreement with a recent study using molecular dynamic simulations of KcsA, in which it was suggested that structural constraints imposed by the channel protein are not important [19]. The authors of the simulation study proposed that selectivity is a function of local chemical interactions provided by a relatively unstructured carbonyl oxygen selectivity filter (described as liquid-like), which creates a proper electrostatic environment for K^+^. Local interactions between carbonyl oxygen atoms and K^+^ ions are certainly important, but the data presented here would seem to suggest that the protein structure is actually very important in the creation of size-constrained (does not mean rigid) ion binding sites. The recent structure of a cation-selective channel, called the NaK channel, reinforces this same conclusion [20]. Its selectivity filter contains two binding sites that are chemically identical (in terms of the coordinating oxygen atoms) to sites 3 and 4 in a K^+^ channel, and yet these sites are not very selective for K^+^ over Na^+^, apparently because the surrounding protein atoms are different and thus impose different constraints upon the sites.

It is useful to place selectivity in the K^+^ channel in the broader context of ion selectivity by synthetic host molecules. Cram, Lehn, and Pedersen demonstrated that ion selectivity could be achieved by constraining the size of the binding site to match the size of the ion [21–23]. We suggest that the K^+^ channel achieves selectivity by the very same principle, using the protein structure to create carbonyl oxygen-based ion- binding sites that are appropriately sized for K^+^ and not for Na^+^. In the K^+^ channel, we observe a new level of complexity added to this principle of size selectivity: the presence of K^+^-selective sites 2 and 3 is coupled to a specific conductive conformation of the selectivity filter. Consequently, K^+^—the ion that is to be conducted—stabilizes the conductive conformation. The K^+^-dependent conformational change represents a form of selectivity, because in the absence of K^+^ (i.e., in the presence of only Na^+^, which should not be conducted), the filter goes into a nonconductive conformation.

## Materials and Methods

### KcsA preparation and purification for ITC.

KcsA was expressed and purified as described previously [2]. The A98G mutant of KcsA was used in all ITC measurements, because equilibration was reached more quickly following each ionic solution injection. This mutant is referred to as wild type in this study. KcsA was cleaved with chymotrypsin and the tetramer purified over a Superdex 200 column equilibrated with 50 mM Tris, pH 7.5, 20 mM KCl, 100 mM NaCl, and 5 mM DM (n-decyl-β-d-maltopyranoside). The purified protein was concentrated to 10 mg/ml, extensively dialyzed against the desired buffer, and diluted just prior to ITC experiments. Protein concentrations were determined by absorbance at 280 nm where 1 optical density (OD) = 0.4 mg/ml. KcsA is stable in these conditions and was reused after exchanging it into the appropriate buffer.

### Isothermal titration calorimetry measurements.

Measurements of the enthalpy change (ΔH°) upon ion binding to KcsA were performed using a VP-ITC MicroCalorimeter (MicroCal; http://www.microcal.com). Any given experiment was carried out at a constant temperature (±0.005 °C) within the range of 21 °C to 23 °C. The sample cell (*V* = 1.3628 mL) was filled with a solution containing 25 mM HEPES, pH 7.5, 100 mM NaCl (except experiments done with 100 mM LiCl), 5 mM DM and 71–142 μM KcsA. The injection syringe was filled with a mono- or divalent salt ligand solution containing 25 mM HEPES, pH 7.5, 100 mM NaCl (or 100 mM LiCl), 5 mM DM and 15–100 mM XCl_n_, where X is the desired ion. All solutions were filtered and degassed prior to use. Twenty to thirty injections of 3–10 μL of ligand solution were titrated into the KcsA protein solution while stirring at 500 rpm. The heat change of each injection was integrated over 10 min for monovalent ion solutions and 20 min for BaCl_2_ solutions, both of which are uncharacteristically slow for simple ion binding, suggesting a slow equilibrium between the nonconductive and conductive conformations (see next section for model). Protonation during ion binding was assessed using solutions containing 25 mM (Na-phosphate or Tris) buffers, pH 7.5 instead of 25 mM HEPES, pH 7.5 in both the cell and syringe (Figure S2).

### Ion-binding model.

**Figure 7:**
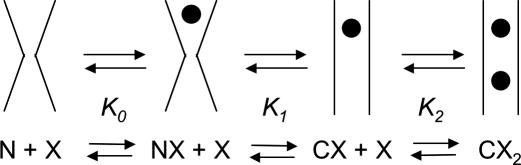
Ion-Binding Model The four-state ion-binding scheme is shown, from left to right: non-conductive conformation without an ion (N), non-conductive conformation with a single ion (NX), conductive conformation with a single ion (CX), and conductive conformation with two ions (CX_2_) with equilibrium constants *K*_0_, *K*_1_, and *K*_2_.

The four-state reaction scheme shown in Figure 7 is the simplest description of the ion-binding event that is compatible with our experimental data. Equation 1 describes the probability that the channel is in the CX_2_ state as a function of K^+^ concentration, denoted as [X].

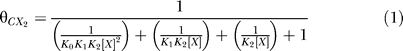



Crystal structures show two distinct conformations of the selectivity filter, nonconductive (NX), and conductive (CX_2_) (Figure 3A). NX is observed at low [K^+^], and CX_2_ is observed at high [K^+^]. Full K^+^ titrations were carried out between these conformations using crystallography, from which we concluded that K^+^ stabilizes the conductive conformation [9].

State N is never observed crystallographically, even at high concentrations of Na^+^, where the filter adopts its nonconductive conformation, which we correspond to state NX. Since we never observe state N, we assume that *K*_0_ is very large, so the quadratic term ([X]^2^) can be dropped and Equation 1 is approximated by Equation 2, a rectangular hyperbola (*n* = 1 binding isotherm) with an apparent *K*_D_ = (1 + *K*_1_)/*K*_1_ *K*_2_ (referred to as *K*_D_ in the text)..

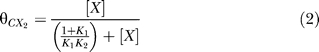



The ITC titration of the wild-type channel is consistent with Equation 2, since the data fit well to an *n* = 1 binding isotherm, and attempts to fit *n* ≠ 1 do not significantly improve χ^2^. One might wish to argue that the equilibrium process N to NX could be coming in to the mechanism, but is simply not detectable as a significant deviation from *n* = 1. Such an occurrence would not change the conclusions of this study.

State CX is also never observed but is included, because we know from crystallography that the pore goes from its nonconductive conformation, with one ion in it at the beginning of a K^+^ titration, to its conductive conformation, with two ions in it at the end. Mutation M96V prevents CX_2_ (filter remains in NX) even at high [K^+^] (Figure 3B). Crystallographic ion occupancy studies show that NX harbors an ion near one of its entryways (referred to as binding sites 1 or 4) but not in the middle (at sites 2 or 3), whereas CX_2_ harbors an ion near one of its entryways (sites 1 or 4) and in the middle (sites 2 or 3) (Figure 3A) [9]. Thus, in the reaction scheme, the transition governed by equilibrium constant *K*_1_ describes a conformational change of the filter from NX to CX and the transition governed by *K*_2_ describes the entry of a single K^+^ into the middle of the conductive filter (to occupy site 2 or 3). Because only states NX and CX_2_ are observed experimentally, we assume that *K*_1_ favors NX and that *K*_2_ favors CX_2_.

According to this scheme, upon addition of K^+^ in our calorimetric titration the formation of the CX_2_ state occurs in two steps. We make no assumptions about the detailed origins of the heat generated (i.e., which steps contribute). However, it is an important observation that the mutation M96V prevents the NX to CX_2_ conformational transition as documented by crystallographic experiments (Figure 3B), and it also prevents the exchange of heat in the ITC experiments (Figure 3D). In essence, the heat serves as a signal for the nonconductive-to-conductive transition, and thus enables us to investigate how different ions react with the filter under equilibrium conditions.

### Fitting the ITC data.

The data were fit in Origin to the equations provided in [8] (fix *n* = 1). A constant background was subtracted because the 100-mM NaCl in the cell and syringe created an environment where the heat of diluting the ions from the syringe is constant; an example of this can be seen both in Figures 2D and 3D, where ions do not bind to KcsA. This background was determined from the integration of the final injections and the minimization of χ^2^ for the overall fit. Ideally, to fit *n, K,* and *H* as independent variables, where *n* is the number of binding sites, *K* is the association constant, and *H* is the enthalpy of the reaction, the product of the association constant and the concentration of protein in the cell (termed “c”) should be within the range of 5–500 (see Micro Calorimetry System ITC manual and [8]). If c < 5, such as in our case where c < 0.3, it is still possible to fit *K* and *H* if outside knowledge of the stoichiometry of binding is known and if the binding sites are at least nearly saturated during the titration [24]. The fit and error to the fit for *K* and *H* for Figure 2B are *K* = 2440 ± 150 M^−1^ and *H* = −1402 ± 49 cal/mol, indicating an excellent fit of the data to the model. At least three full titrations (with similar errors to the fit) of each ligand condition were collected, with their means and standard deviations of the mean reported in Table 1.

### ITC measurements with high KCl (wild-type and M96V KcsA).

No K^+^ ion binding to M96V KcsA was seen in the experimental range described above with up to 35 mM KCl in the syringe (unpublished data). However, it is possible that K^+^ would bind M96V with a lower affinity than wild type, requiring a higher [KCl] solution in the syringe. The experiments in Figure 3C and 3D were performed with a sample cell solution containing 25 mM HEPES, pH 7.5, 250 mM NaCl, 5 mM DM and 142 μM KcsA, and 25 mM HEPES, pH 7.5, 400 mM KCl, and 5 mM DM in the injection syringe. The different salt concentrations were necessary to compensate for the heat of diluting the 400 mM KCl in the syringe, which when injected into a cell containing 100 mM NaCl, dominated the enthalpy from ion binding to the wild-type channel. To reduce this dilution effect, the NaCl concentration in the cell was adjusted to counterbalance the KCl in the syringe until significant binding of K^+^ to wild-type KcsA was observed, which occurred at 250 mM NaCl.

### Co-evolution of positions in K^+^ channels.

An alignment was created from 404 K^+^ channel sequences that were collected from the nonredundant database using PSI-BLAST (e-score < 0.001) [25]. The statistical conservation and coupling between positions were calculated as described previously [26]. The network of co-evolving positions was determined from an analysis that seeks self-consistent clusters of positions that statistically co-vary with one another [27]. K^+^ channels have one self-consistent cluster, which is mapped as the co-evolving positions in Figure 4B.

### KcsA-Fab crystal preparation.

The KcsA-Fab complex was prepared and purified as described [2]. KcsA-Fab in Na^+^ was obtained by extensively dialyzing the purified complex against a buffer containing 50 mM Tris, pH 7.5, 150 mM NaCl, and 5 mM DM over a period of 48 h. The Ba^2+^-containing KcsA-Fab complex was prepared by adding BaCl_2_ (to a 5 mM final concentration) to the KcsA-Fab in Na^+^ just prior to setting up the crystal trays. The KcsA(M96V)-Fab buffer solution contained 50 mM Tris, pH 7.5, 300 mM KCl, and 5 mM DM. Crystals were grown at 20 °C by the sitting-drop method by mixing an equal volume of concentrated KcsA-Fab complex (∼12 mg/ml) with a reservoir solution containing 21% PEG400, 50 mM magnesium acetate, and 50 mM buffer (HEPES, pH 7.0 for Na^+^ crystals or 50 mM MES, pH 6.2 for Ba^2+^ crystals). Crystals were cryoprotected in a single step by increasing the PEG400 concentration in the reservoir solution to 40%, followed by re-equilibration for 1–2 d. All crystals were frozen in propane and stored in liquid N_2_.

### Protein crystallography.

Data were collected at station X25 of the National Synchrotron Light Source (Brookhaven National Laboratory, Upton, New York, United States) and at station A1 of the Cornell High Energy Synchrotron Source (Ithaca, New York, United States). The data were processed with Denzo and Scalepack [28]. The structures were solved by molecular replacement using the high K^+^ KcsA-Fab structure (Protein Data Bank (PDB)code 1K4C) as a search model in molrep [29]. The models were refined by manual rebuilding using the program O [30] and several cycles of minimization and B-factor refinement using CNS [31]. A random 5% of the reflections were excluded from the refinement to calculate *R*_free_. Anomalous Fourier difference maps were calculated for the Ba^2+^ structure in CNS using a model that excludes the bound ions and selectivity filter residues 74–80.

## Supporting Information

Figure S1 Structures of KcsA with Various Ions Bound within the Selectivity Filter (A–E) 2Fo-Fc electron density maps (contoured at 2 sigma) show the selectivity filter of two diagonally opposing subunits with bound ions. (A) The collapsed structure (from PDB 1K4D) probably has Na^+^ bound and is modeled as such in this panel. This result is based on a Na^+^ complex KcsA structure that we have deposited into the Protein Data Bank (PDB 2ITC), which shows that the selectivity filter exists in the collapsed conformation when Na^+^ is the only monovalent cation present.
(B) This conductive K^+^ structure is from PDB 1K4C.
(C) This conductive Ba^2+^ structure is from this paper.
(D) This conductive Rb^+^ structure is from PDB 1R3I.
(E) This conductive Cs^+^ structure is from PDB 1R3L.
(2.6 MB PNG)

Figure S2 Enthalpy of Ion Binding as a Function of Buffer Ionization Enthalpy Δ*H*° of K^+^ ion binding to wild-type KcsA was measured in the presence of phosphate, HEPES, and Tris buffers at pH 7.5. The buffer ionization enthalpy is taken from [32]. Each filled circle represents one experiment and the least-squares fit to the mean is shown, where the slope is the number of H^+^ ions bound per K^+^ ion bound and the ordinate-intercept is the intrinsic enthalpy of K^+^ ion binding. There is little to no enthalpy contribution from buffer ionization during K^+^ ion binding to KcsA (*n*_H_ = −0.08).
(45 KB PNG)

Table S1 Data Collection and Refinement Statistics for Ba^2+^-KcsA complex and M96V KcsA mutant Values in parenthesis indicate statistics from the outer shell only.
(194 KB PNG)

### Accession Numbers

Structure coordinates and structure factors from the KcsA in BaCl_2_, KcsA in NaCl, and KcsA(M96V) in KCl crystals have been deposited in the Protein Data Bank (http://www.rcsb.org/pdb) with accession ID codes 2ITC, 2ITD, and 2NLJ.

## Abbreviations

ITC: isothermal titration calorimetry
KcsA: Streptomyces lividans K^+^ channel
